# Asiatic Cholera—Its Pathology, Treatment, and Prophylaxis

**Published:** 1873-09-15

**Authors:** M. W. Wood


### ASIATIC CHOLERA—ITS PATHOLOGY, TREATMENT, AND PROPHYLAXIS.

A PAPER READ BEFORE THE CHICAGO SOCIETY OF PHYSICIANS AND SURGEONS, AUGUST 11TH, 1873, BY M. W. WOOD, M.D.

*Mr. President, and Gentlemen:*

In presenting for your consideration these remarks upon the subject of cholera, I must at the outset disclaim any pretension to originality.

The literature of the subject is very voluminous, and is of such variety as to confuse and to mystify the cursory reader. On no one question in the whole science of medicine are there so many seeming discrepancies—so many almost diametrically opposed theories—each claiming to be based upon observed facts. Even the same facts have received opposite and distinct interpretations; and theories based upon unsustained assertions, or unsupported save by analogies, have been accepted as laws, losing sight of the fact that analogies are not proofs. So that much time is necessary to compare these, to obtain from them the deductions essential to the proper understanding of the question.

I have, therefore, from such means as were at my command, prepared this paper as an exposition of the present authoritative views of the profession upon the subject. In this article there will of course be much to criticise—much that will not stand by the aid alone of any one of the standard (?) theories of cholera.

I will give the deductions obtained from all the facts which have been attainable by me; the statistics as far as given; opinions and facts as to the various plans of treatment; the main points accepted and proven in the pathology; concluding with some remarks upon the prophylaxis of the disease. The history, and the points of the differential diagnosis, have been so often and so fully given, that their repetition is needless. In passing the subject of the etiology of the disease, it may be stated that *among our best authorities there is perfect harmony in this:* that there is necessary the occurrence of a *specific* cause, which, acting in conjunction with certain local influences, produces the disease; and that these local influences determine the boundaries of the districts affected.

The specific cause—the exact nature of which is yet undetermined—is, as Niemeyer* says, "whatever its nature or origin," not indigenous. And he further says, that all the cholera epidemics are due to what he has been pleased to term "the exotic parasite."†

Dr. Geo. Johnson ‡ says, "the exact nature of the cholera poison, as of every other morbid poison, is unknown; but it certainly has a very close affinity with filth;" and it is a well-known fact that the disease occurs first, last, and with the greatest mortality, among the lower orders of the community. And the concurring causes—several of which are present in every epidemic—may be briefly stated to be: impure water, i. e., that contaminated by decomposing organic matter, improper food, privations, emanations from decomposing animal refuse, intemperance, mental or bodily fatigue, comparative lowness of situation, and last but by no means least, want of cleanliness.

It is believed that the disease is propagated principally by means of the dejections; that these dejections are not at first as poisonous, but that after they have been subjected to atmospheric influences for a longer or shorter time, or, in other words, have been suffered to decompose,§ "the poison germs are developed or vitalized;" and various experiments have been conducted, which have given results in favor of this, as those recorded by Watson,§§ where pieces of filtering paper, soaked in the cholera dejections which had been suffered to decompose, were fed to white mice, 88 per cent. of whom were affected by cholera; and of these 14 per cent. died, with the post mortem appearances of cholera in the human subject. So that when we read of the negative results of other experiments, as those that Hartshorne quotes, ‖ where Dr. Foy and ten other enthusiasts, at Warsaw, inoculated themselves with the blood of cholera patients, and tasted their dejections, without receiving the disease, we are led to believe that the dejections were not sufficiently decomposed!

That more persons are attacked in the early morning than at any other time in the twenty-four hours, is easily enough explained,‖‖ when we recollect that the temperature of the human body is at that time lowest, and that the poison is necessarily most condensed in the lower strata of the atmosphere.

The intestinal discharges will next claim our attention, and the subject is one of considerable importance, as we shall see.¶ "One of the first things to arrest our attention is

* Pract. of Med., 8th ed., p. 643.

† Op. cit. ‡ Med. Times and Gaz., Feb. 3d, '72.

§ Sir R. Martin. §§ Pract. of Phys., p. 923.

‖ Chol., etc., Phil., 1866.

‖‖ Dr. Goodeve. Reynolds' Pract., art., "Cholera."

¶ Dr. Goodeve, op. cit.

the striking difference between the amount of the cholera evacuations and those of sporadic cholera, cholerine, choleraic diarrhœa, or cholera morbus, all of them diseases likely to be confounded by the superficial observer. The amount is frequently 80 to 100 or 150 oz. during the evacuation stage." After the feculent portions, that is, the contents of the large intestine, have passed, they are without smell. Their composition has been carefully studied, chemically and microscopically. Dr. Goodeve says* "the cholera stool separates, upon standing, into a thin fluid portion and a small but variable amount of sediment. The sediment consists of granular matter—small bodies like nuclei—granular cells, some resembling pus cells, and some squamous epithelium. The fluid portion is of specific gravity 1005 to 1010—seldom above 1012; it is faintly alkaline or neutral in reaction; contains but little albumen, and consists chiefly of the water of the blood, with saline matter."

Beale says,† "The stools of cholera patients are remarkable for the large quantity of cylindrical epithelium which they contain."

Frey, in his late work on "The Microscope," ‡ says: "We mention, finally, the cholera stools. The rice-water-like dejections in this disease contain very large numbers of mucous corpuscles; but on the contrary, only very little cylindrical epithelium."

In the last report of the British Army Medical Department, in the paper by Dr. Parkes, on Hygiene, there is a report by Dr. Douglass Cunningham, of Calcutta, on Pettenkoffer's theory, and the microscopic character of cholera discharges, being the result of the microscopic examination of one hundred cholera evacuations.§ "In only four of these was unmistakable epithelium to be found, and in only *one* was it abundant. Red blood cells were present in fourteen cases, infusoria (exclusive of bacteria) in sixty-six, in the form of cercomonads, peculiar monadiform bodies, and distinct amœbæ; these are fully described and figured. With respect to the amœbæ, it is pointed out how difficult it is to draw the line between them and the human cells with amœboid movements like the white blood cells. In the cholera stools they move and increase by division; and when in rows often resemble Hallier's macroconidial chains. *They* are soon destroyed in the stools. On no occasion has any transition from the monadiform bodies into amœbæ been noted. The bacteria and vibriones, and the micrococcus and leptothrix of Hallier are carefully described; in fresh evacuations they have been found in a minimum of development. Recognizable fungi were only found in three of the one hundred evacuations. The most careful cultivation did not produce the cholera series of Hallier. Aspergillus was most commonly produced; penicillium was also seen, but less frequent than aspergillus. The following are the conclusions as to fungi: 1. Distinct fungal elements are not common in, nor characteristic of, cholera evacuations. 2. There is no special tendency to the special development of such elements, or their results, in choleraic, as compared with other decomposing materials. 3. There is no special form of fungus peculiar to choleraic materials. 4. The forms of fungi which appear as the result of the cultivation of choleraic material, with or without the addition of other media, are those which are commonest in other decomposing organic substances at the same season, and under similar circumstances.

"After considering the fungi, Dr. Cunningham passes on to a very careful description of 'Oval and Circular Cells,' which, with a gelatinous or molecular basis really constitute the bulk of the white flocculent deposit of most choleraic evacuations. These cells occurred in eighty-seven of the one hundred evacuations, and in a majority of instances formed the most characteristic features of the sediment.§§ Such are the microscopic examinations of cholera dejecta by Dr. Cunningham, and they are in excellent accord with the observations of his colleague, Dr. Lewis; and if they retain their health, and are permitted to continue their researches, the 'oval and circular cells' will form the subject of renewed investigation by both observers, and eventually much of the mystery of cholera may be cleared up."

In the consideration of the post mortem appearances, I shall only give those upon which all of the careful observers of numbers of cases agree, leaving the occasionally noted lesions entirely out of the question. The blood is thick, black (or very dark), and viscid, or treacly, as it has been termed, and from which no serum will separate on standing. This dark blood, however, brightens on exposure to the air, or to the contents of the small intestine.‖‖ The capillaries of the surface of the body are empty, the blood being chiefly found in the viscera, and notably on the arterial side of the pulmonary circulation. The left side of the heart is empty, or nearly so; at most containing but little black blood, and a few coagula. The skin is wrinkled and

* Op. cit. † Microscope in Med., p. 285.

‡ The Microscope, and Micros. Technology, p. 453.

§ Brit. Army Med. Rep., 1870.

§§ Ibid. ‖‖ Niemeyer, Practice of Med.

blanched*, and when pinched up, it stands in folds. The eyes are shrunken, nose pointed, and cheeks hollow, as after great emaciation. In the stomach there are no constant changes. Generally, however, the mucous membrane is edæmatous; and, as elsewhere throughout the chylopoietic viscera, there is what Niemeyer terms "excessive venous hyperæmia.** The liver has, as its most constant change,† aside from this venous congestion, that it is small, friable,†† and is easily separable from its serous envelope, which is sometimes wrinkled like the skin. The kidneys present about the same changes as the liver. Spleen congested; lungs remarkably dry,††† and free from hypostasis and œdema. The cerebral veins and sinuses are distended, but the substance of the brain dry and hard. The bladder is empty and strongly contracted.

We now come to *the constant lesion;* and I will give a few authorities, using their language. Dr. B. J. D. Irwin,‡ Surgeon U. S. Army, gives as the result of several autopsies during the last epidemic:‡‡ "The mucous membrane at the upper part of the small intestine was blanched, the lower part congested, with erosion of the mucous surface in patches, the intestine near the ileo-cœcal valve intensely inflamed."

Niemeyer‡‡‡ says: "The mucous membrane and the whole intestinal wall are swollen and relaxed; that of the small intestine being finely injected, especially near the valve, and growing less so as we pass upward; the large intestine does not show any constant changes, and the jejunum is variably, if at all, affected."

Dr. J. B. Brown, Surgeon U. S. Army, says:§ "The invariable lesion appearing to be active inflammation and swelling of the solitary and agminated glands of the ileum, with great injection of the points of the solitary glands."

Prof. Stille,§§ in his recently published lecture on "Epidemic Cholera," says: "The important appearance in the intestine being the great loss of epithelium, which is either entirely removed, or detached, adhering loosely."

Niemeyer,§§§ also makes allusion to the paresis of the heart as constituting one of the terrible symptoms of cholera asphyxia: "It only depends on the intensity, and particularly on the extent, of the denudation of the intestinal mucous membrane, whether symptoms of paralysis of the heart shall develop, and whether the loss of water from the blood shall prove dangerous."

Thus it will be seen that the real trouble is two-fold: *Hæmatic and Neurotic.* Hæmatic, in that peculiar disorganizing changes undoubtedly take place in the blood. Neurotic, in that there is an especial disturbance in the innervation of the gastro-intestinal mucous membrane. This disturbance, however, differing in character and degree in the part supplied by that beautiful network of arched branches, the *vaso intestini tenuis*, and to which is distributed a large branch of the superior mesenteric plexus, from the other parts to which are distributed the remainder of the great solar plexus.

In what does this disturbance consist? In experiments recorded in the American edition of Ranking's Abstract,‖ it was shown that extirpation of the cœliac plexus produced hyperæmia of the intestinal mucous membrane; and in the IVth Vol. of "Flint's Physiology,"‖‖ we find: "Since the publication of our volume on secretion (Vol. III.), Dr. Moreau has published a series of observations upon the secretion of liquid in the intestinal canal, which are peculiarly interesting in their bearing upon the sudden occurrence of watery diarrhœa. In these experiments the abdomen was opened in a fasting animal, and three loops of intestine, each from four to eight inches long, were isolated by two ligatures. *All* of the nerves passing to the middle loop were divided, taking care to avoid the blood-vessels; the intestine was then replaced, and the wound in the abdomen was closed with sutures. The next day the animal was killed; the two loops, with the nerves intact, were found empty, as is normal in fasting animals, and the mucous membrane was dry; but the loop with the nerves divided was found filled with a clear alkaline liquid, colorless or slightly opaline, which precipitated flocculi of organic matter on boiling."

If, now, in cholera, the vaso-motors are paralyzed by the influence of the poison in the blood, how plain the whole series of manifestations becomes! The dilated capillaries, enlarging the stomata, permit the escape of the more fluid portion of the blood. And that they are dilated we have the evidence of Beale,‖‖‖ who says: "They are distended to three or four times their ordinary diameter, so that the smallest vessels are injected very easily, and a very rapid transudation of fluid through the capillary walls can easily be made to take place after death." What must necessarily result? The spissitude of the

* Niemeyer, op. cit. ** Op. cit., p. 728.

† Dr. Brewer. Cholera Circular; Circ. No. 1, Surg. Gen'l's Office, 1868.

†† Dr. B. J. D. Irwin, Chol. Circ.

††† Niemeyer, op. cit. ‡ Chol. Circ., op. cit.

‡‡ That of 1867. ‡‡‡ Op. cit. § Chol. Circ., op. cit.

§§ Med. Times, July 12th, '73. §§§ Op. cit.

‖ No. 26, p. 288. ‖‖ p. 434.

‖‖‖ Peters, op. cit., p. 184.

blood being increased, it no longer circulates as rapidly as formerly, but sluggishly and with difficulty.

That the stagnation must necessarily result from the transudation is evident, as the blood corpuscles cannot pass through the capillaries except when separated by the serum. That the respiratory function is imperfectly performed is evinced by the lessened expiration of carbonic acid. The *oxygen carriers* now no longer performing their functions properly, the blood becomes black, does not change from venous to arterial, hence the decrease of temperature, and as well the non-action of the secernent glands. Sir Wm. Gull,* in 1854, in speaking of the pathology of cholera, used this expression: "*The depression of capillary power*, the extreme exhaustion of the *great ganglionic nerve centers of the abdomen*, are peculiar to cholera, and give it an individuality."

That the cerebro-spinal nervous system also participates in the production of the symptoms, is the evidence of many careful observers.

Dr. W. H. Lyne, of Livonia, Louisiana, in a communication to the New Orleans *Medical and Surgical Journal*,** states that having noticed tenderness upon pressure over the spinal column in a case of cholera, he had subsequently seen about one hundred cases, of which only one was without this symptom.

Dr. H. M. Kirke, Act. Ass't Surg. U. S. Army, † who will be quoted further on, has given some interest to an opinion that the spinal cord is implicated. On these, as facts, are based theories of treatment, as Chapman's Ice-bags, and two others to be noted.

Spasms of voluntary muscles are everywhere noticed as symptoms; and not due to non-oxidation alone, as they occur before the circulation is much interfered with. The range of the plans of treatment proposed seems very extensive; but with few exceptions (notably Johnson's), they are based upon the one idea of arresting the discharges, and are generally prefaced with a confession that our art is powerless, and that anything giving a promise is justifiable.

The results of the faithfully-carried-out plans have shown that *many of them were powerless* in influencing the mortality favorably.

I will give you some opinions, as to treatment, from different authorities. All have a bearing.

Johnson himself says,†† speaking of the diarrhœa which ushers in the disease, "The best treatment for the diarrhœa is that which most speedily arrests it without subsequent ill effects."

Prof. Flint, sr., ‡ says, "Prior to collapse the paramount object is the arrest of the intestinal effusion."

Prof. Stillé‡‡ says, "The stage of diarrhœa is the only stage of the disease in which treatment makes a sensible impression upon the mortality."

Dr. Goodeve§ says, "The discharges should be checked, if possible. I believe the great object of treatment is to restrain the passage of the exudation from the blood into the intestine; and the remedies used for this check the vomiting and purging; and this checking, together with the pulse, indicates the amount of interference of the treatment with the disease." And after laying down a plan of treatment, says: "If these medicines check the discharges, all danger will probably be over in a few hours."

Sir Thomas Watson‖ says: "I venture to advise you — supposing the disease should appear, or whenever in the autumn a suspicion arises that cholera is present in the community — not to try, in cases of diarrhœa, to carry off the presumed offending matter, but to quiet the irritation and stop the flux as soon as you can."

As our time is too short to discuss the merits of all of the various plans of treatment, I shall take up only the most prominent.

Johnson's treatment is so familiar that it may be passed over without comment, save this, it has been, according to statistics, *eminently unsuccessful.*

Dr. Grove's treatment, lead and opium in pill, may be dismissed as unimportant.

Dr. Ayers' opium and calomel, in minute doses and often, is also unimportant in this connection.

Dr. Stevens' salines, and Dr. Macintosh's saline injections, have failed as well to influence favorably the mortality.

The prominent modes of treatment which have succeeded best are those by calomel, alone or combined; by morphia, hypodermically; by strychnia; and by sulphuric acid, and the sulphates of the metals iron and copper.

The literature of the calomel treatment is quite extensive. It has many friends high in authority. But many of those who use it, do so, as Macpherson‖‖ says, "without any par-

* Lancet, June, 1854.

** Vol. 12, p. 24. from Wood's Pract. of Med.

† Chol. Circ., op. cit. †† Med. T. & Gaz., '72, p. 127.

‡ Flint's Pract., art. "Cholera." ‡‡ Op. cit.

§ Reynolds' Pract. of Med.

‖ Watson's Pract., p. 920.

‖‖ Peters' op. cit., p. 142.

ticular reason, but regard it as the whist player's trumps, to be played when in doubt." I will give a little of the evidence of those who have used it.

Dr. T. H. Turner, Ass't Surg. U. S. Army,* in a report upon Cholera, says: "In connection with the treatment, I need only say that many remedies of reputed efficacy have been tried; but I have nothing to say in their favor. Mercurials are the only remedies from which I have learned to expect much, and to them I have resorted early and late in the disease."

Dr. H. M. M. Meadows,† in a paper in the *Lancet* on the use of calomel in large doses, says: "I have treated this year 113 cases of cholera, and have lost only six, and four of those by consecutive fever."

Dr. J. F. Randolph, Surg. U. S. Army, †† says of calomel: "It seems to relieve cramps and vomiting sooner and more effectually than anything else tried; and no unpleasant effects were observed from the peculiar action of the drug."

Dr. J. B. Downey, Act. Ass't Surg. U. S. Army,‡ writes: "The stimulant plan of treatment was a failure; of a number of cases that were attacked, but which did not result in death, it seemed that if the secretions could be unlocked by the influence of mercurials, the discharges were controlled."

Dr. J. B. Brown, Surg. U. S. Army,‡‡ who has had experience in at least two cholera epidemics, writes thus of the epidemic of 1867: § "The treatment followed has been the same as that reported last year; and our additional experience seems to confirm the superior efficacy of the large doses of uncombined calomel over the reported results of the other modes of treatment recognized or recommended.

"I can give no satisfactory explanation of the action of the large doses of calomel in relieving cholera. Perhaps as plausible a theory as any, is one which I have for some time entertained: that the calomel, from the absence of acid in the stomach in cholera, passes nearly unchanged into the intestinal canal, and acts locally and mechanically as a remedial agent upon its mucous surface, denuded as it is of epithelium.

"It is a well known fact that dry calomel applied to a chafed or abraded surface externally, wlll stop the exudation of serum, and promote cicatrization more rapidly than any other known dressing."

These instances are sufficient to show that calomel is held in high regard at present in cholera.

Dr. Lyne,§§ in paper already quoted on spinal tenderness, writes this of his 100 subsequent cases: "They *all* recovered, under a treatment consisting mainly of cupping over the spine, with the subsequent application of sinapisms."

Dr. John Patterson,‖ in an article on the treatment by subcutaneous injection of morphia, writes: "A recent severe epidemic in this city (Constantinople) has given me an opportunity of trying the effect of subcutaneous injection of morphia, on a sufficiently large scale to judge of its value. The first cases were treated with the usual remedies, and with the usual want of success. Completely disheartened at the inutility of the treatment, I determined to try morphia subcutaneously. A most unpromising case was selected. The man, previously suffering from inflammation of the liver, was in deep collapse, pulseless, with rice-water purging, severe vomiting and cramps. I injected $\frac{1}{4}$ gr. morphia acetas. The result was beyond our expectations. In a quarter of an hour the cramps and vomiting ceased; the patient fell asleep; the skin gradually became warm and moist, and the pulse returned. In two hours he awoke, and said he felt much better. The injection was repeated. He again slept for three hours. The reaction was perfect. The same good results followed in almost every case in which it was tried. In ordinary cases, one or two injections of $\frac{1}{4}$ to $\frac{1}{2}$ gr. sufficed. In a few cases, three injections were given; and only twice had I occasion to give four. I do not, of course, maintain that the treatment is a specific against cholera. I only claim for it that its action is more decided than any other treatment I have ever seen or practiced."

Dr. H. M. Kirke, Act. Ass't Surg. U. S. Army,‖‖ writes: "My belief that the extreme prostration, nervous in its character, which always accompanies a violent invasion of the disease, indicated that its force was expended almost altogether upon the spinal cord, suggested to me the use of strychnia, as a powerful and certain excitant of the nervous systems, combined, in the first instance, with an aqueous solution of capsicum,

* Chol. Circ., p. 155. † Lancet, Dec., 1854.

†† Chol. Circ., p. 66. ‡ Chol Circ., p. 22.

‡‡ Chol. Circ., p. 62.

§ Ft. Columbus, N. Y. Harbor.

§§ New Orleans Med. and Surg. Jour.

‖ Med. Times and Gaz., 1-27-72.

‖‖ Chol. Circ., p. 40.

to stimulate the stomach and promote the rapid absorption of the strychnia, as—

℞.—Sol. capsici., f. ʒ iv.

Strychnia, gr. $\frac{1}{10}$.

Fl. sol. haust.

Repeat the strychnia in 15 minutes.

"In all the four cases in which this treatment was adopted the improvement was so immediate and marked, that in a few minutes after the second dose the patient fell into a calm sleep, the cramps being *perfectly*, and the other symptoms very much relieved. The treatment afterward was expectant, and all recovered. In all cases the usual symptoms of cholera were present."

Another remedy, which has done much, and promises much, in cholera, is sulphuric acid. Dr. Macnamara,* in his work on Asiatic Cholera, recommends the use of dilute sulphuric acid, in doses of 15♏, in the second stage.

The celebrated "Austrian Specific" for cholera was composed of sulphuric and nitric acid.

The "British Army Cholera Mixture,"—

℞.—Ol. anisi.

Ol. cajuputi.

Ol. juniperis, aa. f. ʒ ss.

Ether. sulphurici, f. ℥ ss.

Liq. Ac. halleri, f. ʒ ss.

Tinct. cinnam., f. ℥ ij.—M.

which contains as its active ingredient sulphuric acid, is ordered to be kept in store by all the medical officers in India.

Dr. Peters,† in his admirable monograph upon Cholera, says: "Sulphuric acid stops diarrhœa, and relieves pain; it is suited to atonic and pale serous diarrhœa in every stage, and often acts like a charm."

Sulphuric acid forms the active ingredient of all the secret cholera medicines that have been known to be efficacious in the treatment of the early stages of cholera. It has been recommended ‡ by Dr. Cox, Mr. Buxton, and Dr. Fuller, of England; and Dr. Jules Wouves reports 288 cases treated in 1865.

Dr. J. G. Sproston,§ in the *Lancet*, says: "During the month of September I have prescribed for 150 cases, and have not had one fatal case. The plan I adopt is simple. To an adult I give an 8 oz. mixture, composed as follows:

℞.—Ac. sulph. dil., f. ʒ ij.
Syr. papaver., f. ℥ iij., simply to color it.
Liq. ammon. acet., f. ℥ ij.
Aq. menth. pip., f. ℥ ijss.—M.
Sig. f ℥ i. after every loose motion.

And very rarely had I to give a second bottle of medicine."

Thus ends the treatment of cholera.

The statistics are mainly those reported to the Treatment Committee of the Medical Council of the Board of Health for Great Britain, and refer to the epidemic of 1854, in which the deaths in England and Wales were 20,000, and in London alone 10,000.

Taking all grades of the disease, the deaths were: §§

| | | |
|---|---|---|
| Of those treated with eliminants, | $71\frac{07}{100}$ | per ct. |
| " " " " stimulants, | 54 | " |
| " " " " calo. & opium, | $36\frac{20}{100}$ | " |
| " " " " chalk" " | $20\frac{30}{100}$ | " |

The mortality, as far as I have been able to learn, of the treatment by injections into the veins, has been $83\frac{98}{100}$ per cent.

The mortality of the cases occurring in the United States army,‖ during the epidemic of 1866, was $45\frac{07}{100}$; and during the year 1867 it was $46\frac{50}{100}$.

The mortality in 1867, among those treated by Dr. Brown's method, in his own practice, was $51\frac{43}{100}$.

Strange as it may appear, and probable as it is that such have occurred, I have not been able to find a record of a single death from cholera, when treated by sulphuric acid; and I hope yet to show that it has superior claims to our consideration as the most promising treatment.

As conservators of the public health, the medical profession cannot but be concerned in the abatement or prevention of every cause of disease.

Much time has been spent upon, and much written upon, the subject of prophylaxis of cholera. Excellent hygienic regulations have been devised, and are familiar to all. But prophylaxis means something more than observance of the principles of hygiene. Many remedies have been proposed as preventives of the disease; but one of them, however, carries any weight of evidence in its favor; that one is sulphuric acid.

Some years since, in England, a large number of people were protected by this agent during an epidemic. But I have been unable to find an authentic account of it.

Dr. Curtin,‖‖ (Res. Ph. at Insane Dep't Phil. Hos., where the disease occurred in a virulent form) in his late paper on this agent as prophylactic in cholera, in mentioning this circumstance, says: "I was induced to try sulphuric acid by my friend Dr. James F. Wilson. Dr. Wilson had read an article in a British *newspaper*, in which the writer stated that the workmen and their families connect-

* Med. Times, July 12th, '73.

† Cholera, etc., Peters, 1867.

‡ Hartshorne, op. cit.

§ Lancet, Jan., 1854; com'n dated Oct., 1853.

§§ Hartshorne, op. cit.

‖ Chol. Circ.

‖‖ Med. Times, July 12th, '73.

ed with a large factory, had been treated with sulphuric acid as a prophylactic, during an epidemic of cholera; and the result was that not one man, or any of their families, were attacked with diarrhœa, while around them death took its own course. I caught, as a drowning man, at this straw, for everything else had been tried, and yet the disease continued its ravages. What followed the administration of the acid can best be shown by notes taken at the time.

"Prior to August 20th, 1866, there had occurred 17 cases.

| | | | |
|---|---|---|---|
| "August 20th, | 4 | new cases. | |
| " 21st, | 4 | " " | |
| " 22d, | 4 | " " | |
| " 23d, | 2 | " " | |
| " 24th, | 5 | " " | |
| " 25th, | 1 | " case. | Acid given for the first time, in the afternoon. |
| " 26th, | 4 | " cases | Within 12 hours after first administration of the acid. |
| " 27th, | no | " case. | |
| " 28th, | 1 | " " | A woman, who would not take the acid. |
| " 29th, | no | " " | |
| " 30th, | " | " " | |
| " 31st, | " | " " | Acid discontinued. |
| September 1st, | " | " " | |
| " 2d, | 2 | " " | Two days after suspension of the acid. |
| " 3d, | | | Acid resumed, and continued for some time. |

"No new cases occurred, though cases continued to arrive from the city until the first of November. Dr. Wilson gave it to the patients in the Surgical wards during the epidemic; and he informs me that *the cholera visited every department of the Almshouse and Hospital, except the Surgical wards.*" Was it the sulphuric acid which prevented the disease? Dr. Curtin thinks so, for the following reasons:

"'1st. *The prompt disappearance of the disease within twelve hours of the time when the acid was first administered.** The only case arising during its use being an insane woman, who refused to receive it. The acid was stopped on the 31st of August, and two days afterwards two cases occurred. After its resumption, no new cases occurred.

"'2d. *The fatality attending the disease.*—When an epidemic declines from natural causes, it generally becomes less fatal. Of the four patients attacked on the 26th of August, three died, one of them in five hours and one in nine hours after the first symptom.' And the average duration of fatal cases is a fraction over two days.† The two cases on the 2d of September both died, thus showing that the type was none the less fatal."

*Op. cit.

†Registrar Gen'l, Eng., 2 .22½–100ths.

Peters †† also says: "MacCormac put a stop to an epidemic in the Belfast Asylum, by administering a daily dose of f. ʒj. ac. sulphur. dil. in f. ℥j. aq. menth. pip.; and no subsequent cases occurred."

Dr. E. A. Sansom, in the *Med. Press and Circular*,‡ in an article on the Anti-zymotic Properties of Sulphurous Acid, says: "The researches of Chauveau have shown that sulphuric acid is prophylactic and curative in cholera, by means of its anti-zymotic properties."

Dr. Bury,‡‡ in the *Lancet*, states that in the last epidemic of cholera in Paris, where the population was decimated, he made careful and personal investigations, and found that of 32,000 workers in copper, brass and bronze, but 16 died, a mortality of $\frac{1}{20}$ per cent., or five in every 10,000; while the wives and families of these workers suffered as others. And the city of Rio Tinto, § surrounded by copper mines, has never been visited by cholera. So, too, workers in gas works are not very liable to the disease; nor those living in the immediate vicinity. But the atmosphere of these places, as of the copper smelting works, is strongly impregnated with sulphur.

And sulphur, as such, has received strong testimonials as a prophylactic. "Dr. Blacklock,§§ of the Madras British army, proposed it as a curative and prophylactic."

And Dr. J. Grove, ‖ after extensive experience, believes "that a fair and judicious use of this remedy will give results as certain as any within the domain of medicine."

Sulphuric acid acts locally as an astringent; it is also a tonic; and those conditions of the system which most favor an attack of cholera seem to call for just such a remedy.

It is also diuretic. And we know how much need there is always in cholera for a diuretic; but that the time cannot be appropriated by them.

Headland ‖‖ says: "It acts chemically, as an acid, in the blood, and in the secretions. When diluted it is easily absorbed; and meeting in the stomach with an acid or neutral secretion, it passes into the circulation without being neutralized. In small quantity, it is neutralized by the small amount of alkali in the blood. If in large quantity, it may exceed this, displacing and setting free other acids in the blood, and combining with their bases. In all cases it increases the quantity of free acid in the system, and renders the secretions, as the urine, more acid than they

††Op. cit.

‡Feb., 1869. In Braithwaite for July, 1869.

‡‡Lancet, Jan., 1854. Med. T., Phila., Curtin's art.

§Curtin, op. cit. §§Waring, Therap.

‖Waring, op. cit. ‖‖Action of Meds., 352–3.

were before. It is also an astringent, coagulating albumen, and causing contraction of muscular fibre. Though neutralized and combined in the blood, it is free before absorption and after secretion. Before absorption it acts upon the surface of the mucous membrane of the stomach as an astringent, and, after excretion, upon the surface of the mucous membrane of the intestine.

"As it does not pass off by the urine, and is known to act as an astringent in diarrhœa, it must seek this outlet, and, constringing the muscular fibre of the intestinal glands, check their secretion."

Dr. Curtin* says: "That if the acid is administered for a long time continuously, it may give rise to symptoms of intestinal irritation." But though he gave it to the lunatics uninterruptedly for several weeks, no ill effects were observed."

And it is a fact about this acid that its administration may be continued without ill effects for a longer time than any other of the mineral acids, save one.

*Op. cit.

